# Post-Transplant Nivolumab Plus Unselected Autologous Lymphocytes in Refractory Hodgkin Lymphoma: A Feasible and Promising Salvage Therapy Associated With Expansion and Maturation of NK Cells

**DOI:** 10.3389/fimmu.2021.753890

**Published:** 2021-11-05

**Authors:** Fabio Guolo, Paola Minetto, Silvia Pesce, Filippo Ballerini, Marino Clavio, Michele Cea, Michela Frello, Matteo Garibotto, Marco Greppi, Matteo Bozzo, Maurizio Miglino, Monica Passannante, Riccardo Marcolin, Elisabetta Tedone, Nicoletta Colombo, Rosa Mangerini, Alessandra Bo, Maria Rosaria Ruzzenenti, Paolo Carlier, Alberto Serio, Silvia Luchetti, Alida Dominietto, Riccardo Varaldo, Simona Candiani, Vanessa Agostini, Jean Louis Ravetti, Genny Del Zotto, Emanuela Marcenaro, Roberto Massimo Lemoli

**Affiliations:** ^1^ Department of Oncology and Hematology (DIPOE), IRCCS Ospedale Policlinico San Martino, Genoa, Italy; ^2^ Clinic of Hematology, Department of Internal Medicine (DiMI), University of Genoa, Genoa, Italy; ^3^ Department of Experimental Medicine (DIMES), University of Genoa, Genoa, Italy; ^4^ Department of Earth, Environment and Life Sciences (DISTAV), University of Genoa, Genoa, Italy; ^5^ PathologIcal Anatomy and Histology, IRCCS Ospedale Policlinico San Martino, Genoa, Italy; ^6^ Stem Cell Processing Unit, IRCCS Ospedale Policlinico San Martino, Genoa, Italy; ^7^ Blood Transfusion Service and Hematology, IRCCS Ospedale Policlinico San Martino, Genoa, Italy; ^8^ Core Facilities, Area Aggregazione Sevizi e Laboratori Diagnostici, IRCCS Giannina Gaslini, Genoa, Italy

**Keywords:** Hodgkin lymphoma, nivolumab, natural killer cells, programmed cell death receptor 1, NK cell maturation, immune check point, autologous & allogeneic transplantation, CD56

## Abstract

Immune checkpoint inhibitors (CI) have demonstrated clinical activity in Hodgkin Lymphoma (HL) patients relapsing after autologous stem cell transplantation (ASCT), although only 20% complete response (CR) rate was observed. The efficacy of CI is strictly related to the host immune competence, which is impaired in heavily pre-treated HL patients. Here, we aimed to enhance the activity of early post-ASCT CI (nivolumab) administration with the infusion of autologous lymphocytes (ALI). Twelve patients with relapse/refractory (R/R) HL (median age 28.5 years; range 18-65), underwent lymphocyte apheresis after first line chemotherapy and then proceeded to salvage therapy. Subsequently, 9 patients with progressive disease at ASCT received early post-transplant CI supported with four ALI, whereas 3 responding patients received ALI alone, as a control cohort. No severe adverse events were recorded. HL-treated patients achieved negative PET scan CR and 8 are alive and disease-free after a median follow-up of 28 months. Four patients underwent subsequent allogeneic SCT. Phenotypic analysis of circulating cells showed a faster expansion of highly differentiated NK cells in ALI plus nivolumab-treated patients as compared to control patients. Our data show anti-tumor activity with good tolerability of ALI + CI for R/R HL and suggest that this setting may accelerate NK cell development/maturation and favor the expansion of the “adaptive” NK cell compartment in patients with HCMV seropositivity, in the absence of HCMV reactivation.

## Introduction

Hodgkin lymphoma (HL) is a lymphoid malignancy of B-cell origin with a high cure rate (1). However, despite the efficacy of frontline therapy, about 30% of patient are refractory or relapse (R/R) (2). In this subset, standard salvage treatment includes high-dose chemotherapy followed by autologous stem cell transplantation (ASCT) (3). However, in order to be effective, ASCT should be performed in chemo-responsive patients (3–5). Unfortunately, 30-50% of patients treated with salvage chemotherapy fail to achieve at least partial response (PR) and Brentuximab-Vedotin (BV, an anti-CD30 immune-conjugated antibody) is able to induce a response in only 30-50% of cases (6, 7). Indeed, HL patients not achieving at least a partial response after second- or third-line chemotherapy have a poor prognosis (8).

Recently, the clinical application of immune checkpoint inhibitors (CI), in particular the PD-1 targeting antibody nivolumab, has dramatically improved the prognosis of patients with advanced phase solid tumors (9). Nivolumab has also shown promising results in HL patients relapsing after ASCT and is currently approved for this setting. Unfortunately, the complete response (CR) rate is only about 20% and median progression-free survival (PFS) is about 18 months, with no evidence of plateau (7, 8, 10, 11). In this view, several reports have highlighted the importance of patient immune-competence to achieve durable response with anti-PD-1 immunotherapy (12–14). Indeed, ASCT, especially in heavily pre-treated patients, leads to a prolonged and deep immunosuppression (12). In this view, re-infusion of previously cryopreserved unselected lymphocytes has been used to boost T-cell count during radio and chemotherapy in the solid oncology field (15).

Several lymphocyte populations have been shown to play a role in the complex immunological mechanism of CI therapy (9, 16, 17). Notably, Reed Sternberg cells display a low/absent expression of HLA I-II complex thus potentially excluding a major contribute from cytotoxic T-cells in response to PD-1/PDL-1 blockade in HL (18). Natural killer (NK) lymphocytes represent important innate effector cells able to kill tumor cell not expressing HLA class I molecules (19–21). NK cell function is finely regulated by an array of receptors transducing either inhibitory or activating signals (20). Among receptors negatively regulating NK-cell function, a crucial role is played by inhibitory HLA class I-specific receptors. In humans, these receptors include the killer Ig-like receptors (KIRs) family, sensing HLA allo-typic determinants (22), and CD94/NKG2A heterodimer specific for the non-classic, class I molecule HLA-E (23). Moreover, different maturation stages are primarily identified according to the progressive downregulation of CD94/NKG2A and the expression of KIRs and CD16. Notably, CD57 marks only late stages of NK cell differentiation.

The aim of this pilot study was to assess the feasibility of early post ASCT CI therapy supported by unselected autologous lymphocytes infusions (ALI). Our results show the safety and feasibility of the procedure. In addition, preliminary data suggest a good clinical response rate in heavily pre-treated high-risk HL patients. These results also highlight phenotypic/developmental diversities in NK cells maturation of treated patients in comparison with the control group.

The combination of adoptive cell therapy with early CI may therefore represent a promising novel approach for R/R HL patients.

## Materials and Methods

### Study Endpoints

The primary endpoint of this study was to investigate the feasibility and safety of post-ASCT nivolumab immunotherapy with the support of ALI in R/R HL patients.

Secondary clinical endpoint was the assessment of the clinical efficacy of the strategy.

The immunophenotypic evaluation of peripheral blood (PB) lymphocyte subpopulations, performed with pre- and post-adoptive immunotherapy and CI administration, with particular focus on NK cell characterization, was an additional biological secondary endpoint.

### Selection of Patients, Study Population, and Treatment

High-risk HL patients (i.e. patients resistant to first line ABVD or showing early relapse) underwent steady-state unselected lymphocyte apheresis with a target cell dose of 1x10^8^ CD3+ cells/kg. They subsequently received salvage therapies including BV if necessary. Patients failing to achieve CR represented the treatment group whereas responding patients were included in the control group. All patients underwent ASCT conditioned with FEAM chemotherapy (see **Supplementary Materials**). All but three treated patients had anti-HCMV IgG at the time of transplantation. No patient experienced HCMV reactivation/infection. CMV DNA was assessed twice a week during all study period in all patients, performed by standard RT-PCR diagnostic test, and was always found to be undetectable.

**Figure 1:**
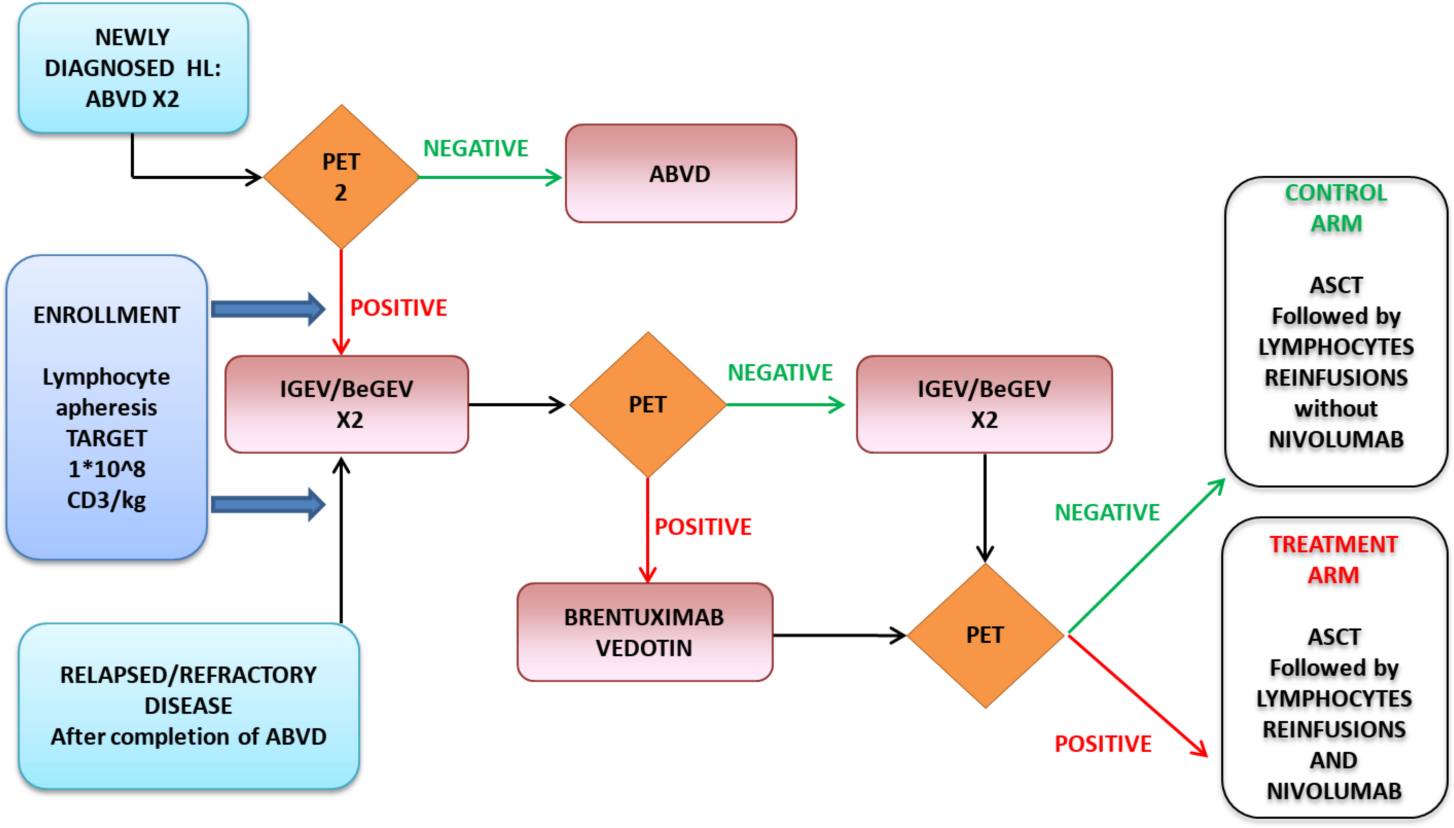
Study Flowchart.

The protocol outline is shown in **Figure 1**. Previous therapies and disease status at ASCT are shown in **Table 1**.

**Table 1:** Clinical characteristics of patients.

	Age at diagnosis	Sex	Stage at diagnosis	Previous Therapies and Response			Disease status at ASCT	Stage at ASCT	Extranodal disease at ASCT
ALL PATIENTS (n=9)	28.5	Female: 5/9	II: 4/9		–		9/9 PD	IV: 9/9	Multiple in 7/9
	(median)	Male: 4/9	III: 1/9						Single in 2/9
			IV: 4/9						
**Patient #1**	26	F	IIB	2xABVD-> PD			PD	IVB	Lung, liver
				2xIGeV-> PD					
				4xBV-> PD					
**Patient #2**	19	M	IIA	2xABVD-> PR			PD	IVA	Lung, pericardium
				2xIGeV-> NR					
				4xBV-> PD					
**Patient #3**	56	M	IIA	6xABVD-> PD			PD	IVA	Lung
				2xIGeV-> NR					
				4xBV-> PD					
**Patient #4**	32	F	IVA	6xABVD-> PD			PD	IVB	Lung, liver
				4xIGeV-> NR					
				4xBV-> PD					
**Patient #5**	22	F	IVB	6xABVD-> PD			PD	IVB	Lung, liver, bone
				3xBeGeV-> PD					
				4x BV-> PD					
**Patient #6**	37	F	IIB	2xABVD-> PR			PD	IVB	Lung, bone
				4xIGeV-> PD					
				4xBV-> PD					
**Patient #7**	31	F	IIIB	2xABVD->PD			PD	IVB	Lung, pancreatic
				4xBeGeV->RP					
				4xBV-> PD					
**Patient #8**	20	M	IVB	2xABVD->PR			PD	IVB	Lung, bone, bone marrow
				4xBeGeV-> NR					
				4xBV-> PD					
**Patient #9**	65	M	IIA	2xABVD->RP			PD	IVB	Lung
				3xBeGeV-> NR					
				4xBV->PD					
**Control #1**	52	F	IIB	2xABVD-> PR			CR	–	–
				4xIGeV-> CR					
**Control #2**	34	F	IIIA	6xABVD-> PD			CR	–	–
				4xIGeV-> CR					
**Control #3**	63	F	IIIA	2xABVD-> PD			CR	–	–
				4xBeGeV-> SD					
				2xBV-> CR					

Nine R/R HL patients failing to achieve at least a PR after three lines of therapy, including BV, and showing persistent disease early after ASCT were included in the treatment cohort and received ALI plus nivolumab 240 mg flat dose, delivered 48 hours after each ALI. Three patients achieving CR before ASCT with either IGeV/BeGeV salvage chemotherapy or third line BV received ALI only without nivolumab, and served as control group. In both groups, the same PB analysis was performed at the same time points (see below for details).

The number of planned ALI for each patient was four. After ASCT, the first ALI was performed at a median of 14 days after stem cell reinfusion (range 12-16), with at least 3 days wash out from the last filgrastim administration. The second ALI was delivered after 14 days, and the other two infusions were performed every 3 weeks.

**Figure 2:**
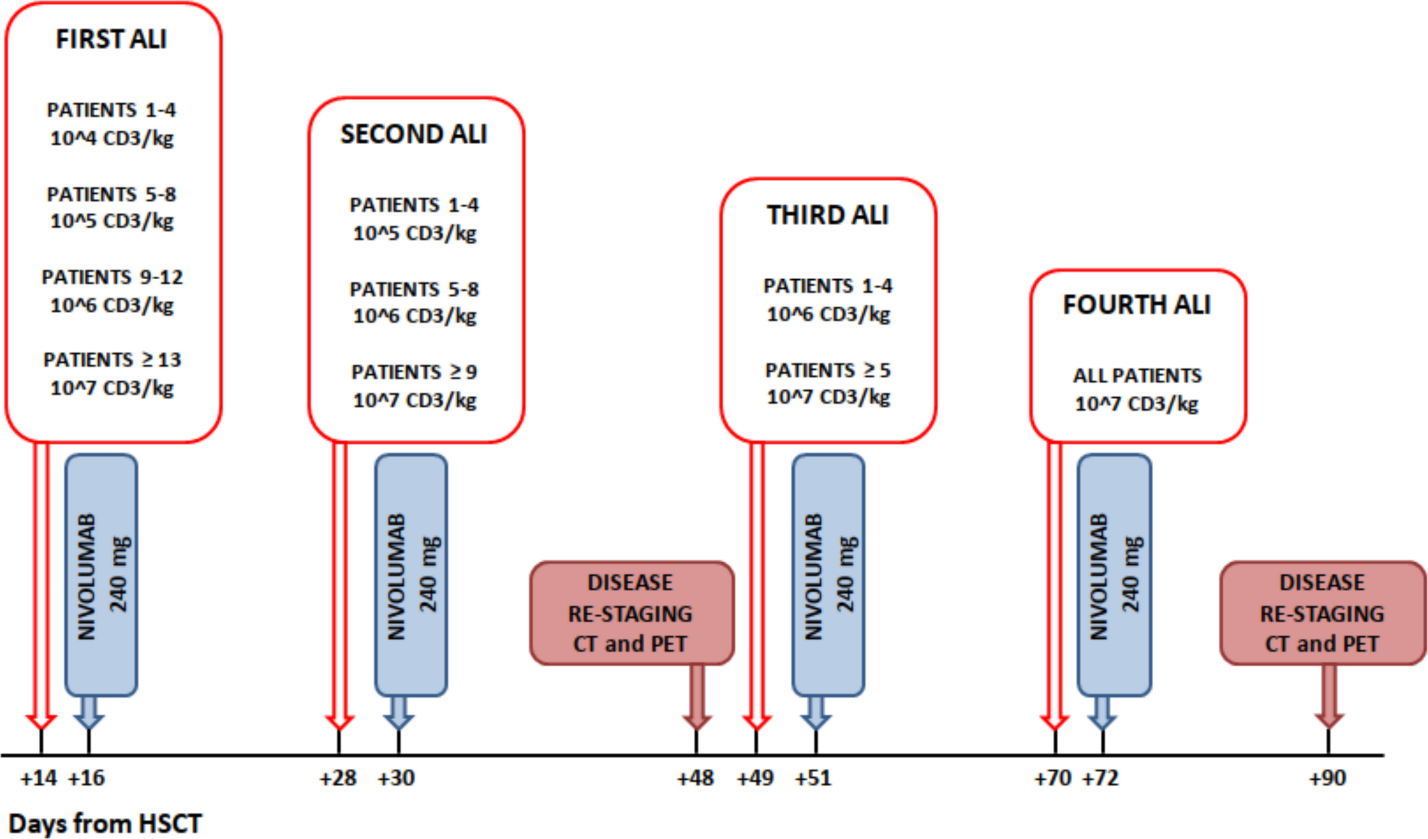
Study treatment.

Although no adverse events with autologous lymphocytes re-infusions were expected, for safety reasons CD3+ dosing was incremental (24), with an increase of 1 log for each of the 4 planned infusion (i.e. from 1x10^4^/Kg up to 1x10^7^/Kg). Furthermore, the starting dose of cycle 1 was planned to increase by 1 log every four patients if no grade >2 adverse events were observed. Thus, the first four patients received 1x10^4^ CD3+/kg in the first infusion and then we escalated the dose to 1x10^5^ CD3+/Kg. The schedule of ALI reinfusion and CI treatment is detailed in **Figure 2**. Assessment of disease response was performed in all patients after the first two cycles of ALI + nivolumab and 21 days after completion of the fourth infusion. Patients achieving CR were offered allogeneic stem cell transplant (HSCT), if feasible.

Adverse events were recorded and graded in all patients according to CTCAE v 5.0.

Commercially available Nivolumab was administrated to all patients according to the indications approved by the European Medicines Agency (EMA).

The study was conducted according to the guidelines of the Declaration of Helsinki. All study procedures were performed as per standard clinical practice: All patients signed informed consent to: data collection and analysis, collection and storage of blood samples for biological studies, lymphocyte apheresis, autologous stem cell transplantation, immunotherapy with CI.

### Lymphocyte Subpopulations Analysis

The phenotypic profile of lymphocyte subpopulations was assessed by flow cytometry (BD-FACSCanto II cytometer) at the following timepoints: 2 hours before and 48 hours after each of the four scheduled ALI and, in the treatment group only, 48 hours after each nivolumab administration.

Single platform absolute counts of major lymphocyte subsets were performed by standard PB immunophenotyping. Briefly, fresh EDTA-anticoagulated PB (50 ul) was stained with the following conjugated monoclonal antibody combination in 8 color multiparametric flow cytometry (FITC, fluorescein isothiocyanate/PE, phycoerythrin/PerCP-Cy™5.5, peridinin-chlorophyll proteins-cychrome 5.5/PE-Cy™7, PE-cyanine dye 7/APC, allophycocyanin/BD™ APC-H7, allophycocyanin-H7/V450, BD Horizon™ V450/V500, BD Horizon™V500: CD3/CD16/CD56/CD4/CD19/CD38/CD8/CD20/CD45 in TruCount tubes. After 10 minutes incubation at room temperature, PB was lysed with 2 ml lysing solution (BD Pharmlyse 1X) for 5 minutes and 250000 lymphocytes were acquired in a BD FACSCanto II cytometer. T lymphocytes (CD3+), T cell subsets (CD3+, CD4+ CD8neg and CD3+, CD8+, CD4neg), B lymphocytes (CD19+, CD20+), NK cells (CD3-, CD16+, CD56+ and CD3-, CD16+, CD56-) were evaluated by using FACS DIVA software. Based on CD45 expression in a CD45 *vs* side scatter (SSC) a leukocyte gate was drawn to include granulocytes (CD45+/high SSC), monocytes (CD45/medium SSC), and lymphocytes (CD45+/low SSC). Results are expressed as leukocytes and lymphocytes counts, % of lymphocytes and % of monocytes of the total leukocytes. Lymphocyte populations are expressed as % of total leukocytes % lymphocytes and absolute counts (N/mmc).

To study the antigenic profile of T and NK cells, PB (2 ml) was bulk lysed with 15 ml lysing solution (BD Pharmlyse 1X) for 10 min, centrifuged at 1500 rpm, washed once with 5 ml PBS-1%FCS, 0,1% NaN3 (PBS) and the pellet resuspended in PBS at 20 x 10^6^/ml. Cells (50ul) were incubated with the following conjugated monoclonal antibody combinations: 1) CD45RA/CD62L/CD3/CD27/CD28/CD8/CD4/PD1, 2) CD45RA/CD62L/HLADR/CD25/CD38/CD8/CD4/PD1, 3) CD57/CD45RO/CD27/CD28/CD8/-/CD45, 4) CD57/CD16/CD3/CD56/-/CD14/-/CD45.

All antibodies had been purchased from Becton Dickinson (San José, CA).

Differential expression of CD45RA, CD62L, CD27 and CD28 was used to identify maturation subsets of CD4 and CD8 T cell populations (CD3+, CD4+ or CD8+) as shown in **Table I-S** (**Supplementary Materials**).

The activation markers CD25, HLADR, CD38 were studied on naïve (CD45RA+, CD62L+) *versus* non-naïve CD8 and CD4 T cells. Dysfunction (previously called “exhaustion”) was studied by the expression of inhibitory receptor PD-1 by gating on the various differentiation subsets based on CD45RA and CD62L expression levels and on activation markers (25).

NK cells (CD56+CD3-/CD56-CD16+CD3-) were further characterized in four subsets: CD56brightCD16neg/dim (immature), CD56dimCD16bright/CD56negCD16bright (mature), CD56dimCD16dim (unconventional NK cells). Based on the expression of NKG2A and KIRs, CD56dim NK cells were then divided into three main subpopulations: maturing (CD94/NKG2A+KIRs-), double positive (CD94/NKG2A+KIRs+), and mature (CD94/NKG2A-KIRs+) NK cells. Within this latter subpopulation, we also evaluated the expression of CD57 as a marker of terminal differentiation. Moreover, we studied the expression of NKG2C on all NK cells to evaluate their “adaptive” phenotype. NK cell subpopulations were analyzed based on a gate strategy already described in detail elsewhere by our group (26). To perform NK cell subpopulation analysis, PBMCs were acquired on a BD Fortessa X20 cytometer and analyzed using a Kaluza software (v.2.1, Beckman Coulter).

### ^18^F-FDG PET/Computed Tomography Acquisition

All patients underwent preparation and FDG PET/computed tomography (CT) according to European guidelines and data were acquired using a 16-slice PET/CT hybrid system (Biograph 16, Siemens Medical Solutions, Knoxville, TN) (27).

All FDG-PET scans were evaluated at Nuclear Medicine Unit of our institution, in keeping with the consensus recommendations, by means of the Deauville 5-point score; thus, sites of residual uptake before allograft were compared with the uptake in the normal mediastinal blood pool and the liver as follows: Score 1, no uptake; Score 2, uptake ≤ mediastinum; Score 3, uptake > mediastinum and ≤ liver; Score 4, uptake moderately increased above liver at any site; Score 5, markedly increased uptake above liver or new sites of disease (28). A Deauville score ≥4 was considered as FDG-PET positive (28).

### Statistical Methods

Dichotomous variables were compared using the Chi-Square test or by Fisher’s exact test when necessary. Continuous variables were compared using Student’s T test, or if normal distribution could not be confirmed, by Wilcoxon’s rank test (29).

Overall Survival (OS) was calculated from the time of transplantation until death by any cause, or last follow-up.

Survival curves were built using the Kaplan Meier method, and univariate survival analysis was performed using the Log-rank test (29).

All statistical analyses were performed using IBMSPSS v22^©^ running on a Debian (Linux) operating system.

**Figure 5C** was plotted using Graph Pad Prism version 8.1.1.

All two-tailed p values < 0.05 were considered statistically significant.

## Results

### Patients

Nine R/R HL patients were treated with ALI + nivolumab in this pilot study. All patients had failed to achieve CR with first- and second-line chemotherapy and progressed during BV therapy. Median age was 28.5 years (range 18-65). PET scan before ASCT showed progressing disease in all patients, with multiple-extra nodal involvement in 7/9. Three additional patients, who had achieved CR with salvage chemotherapy, received ALI alone. Patient’s features are provided in **Table 1**. All patients proceeded to ASCT and achieved complete neutrophil and platelet engraftment after a median of 10 days (range 8-10).

### Feasibility and Toxicity

All but one HL patients achieved the collection of the target cell dose of CD3+ cells 1x10^8^/kg with one apheresis. Patient #8 underwent 2 apheresis. During ASCT, patients experienced the following adverse events correlated to ASCT: fever of unknown origin in 5/12 patients, grade 2-3 mucositis in 6/12 patients, sepsis in 3/12 patients.

No grade 3 or 4 adverse events related to ALI or nivolumab were recorded in the first four patients, so that the following cohort received the first ALI at the increased dose of 1x10^5^ CD3+/kg, without any side effect. In the whole study, no infectious complications or other adverse events were observed during ALI +/- nivolumab therapy. In particular, no patient experienced CMV reactivation/infection and no signs of auto immune disease related to anti PD-1 treatment were recorded.

### Lymphocytes Cytofluorimetric Analysis

Analysis of T-cell subpopulations did not show significant alterations during ALI + nivolumab treatment between treated patients and control group (data not shown).

**Figure 3:**
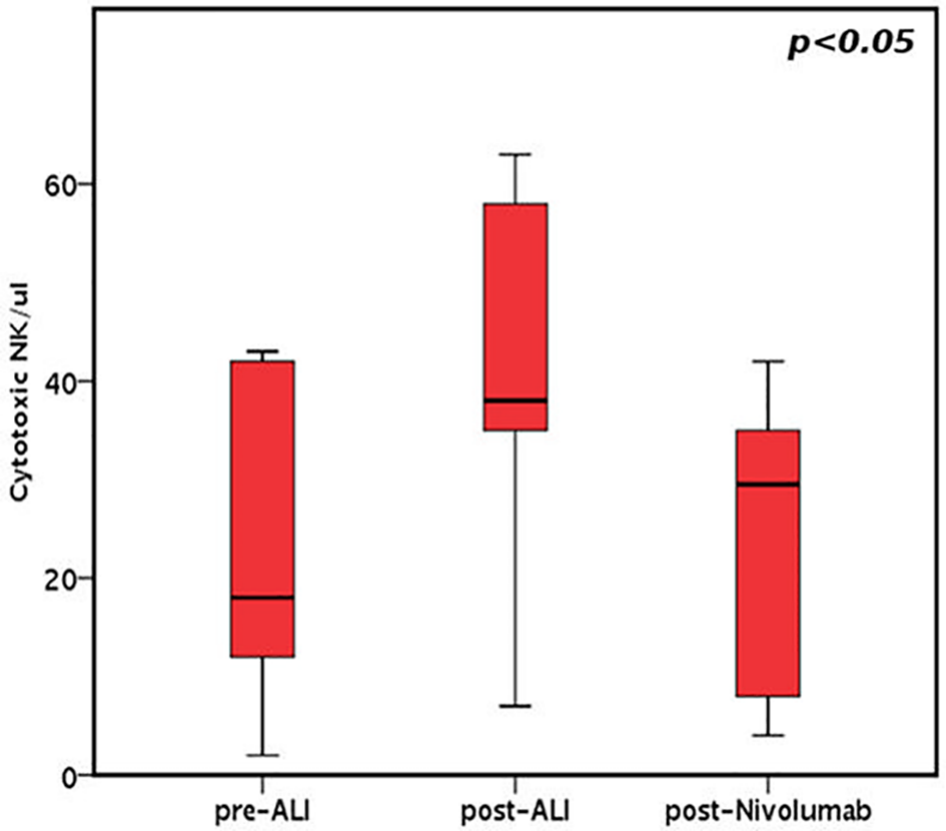
Cytotoxic NK cell/ul before and after ALI in all patients receiving ALI + nivolumab.

On the contrary mature NK cells (i.e. CD56dimCD16bright/CD56negCD16bright; **Figure 3**) showed a significant increase after ALI and after CI (p<0.05 at both time points).

**Figure 4:**
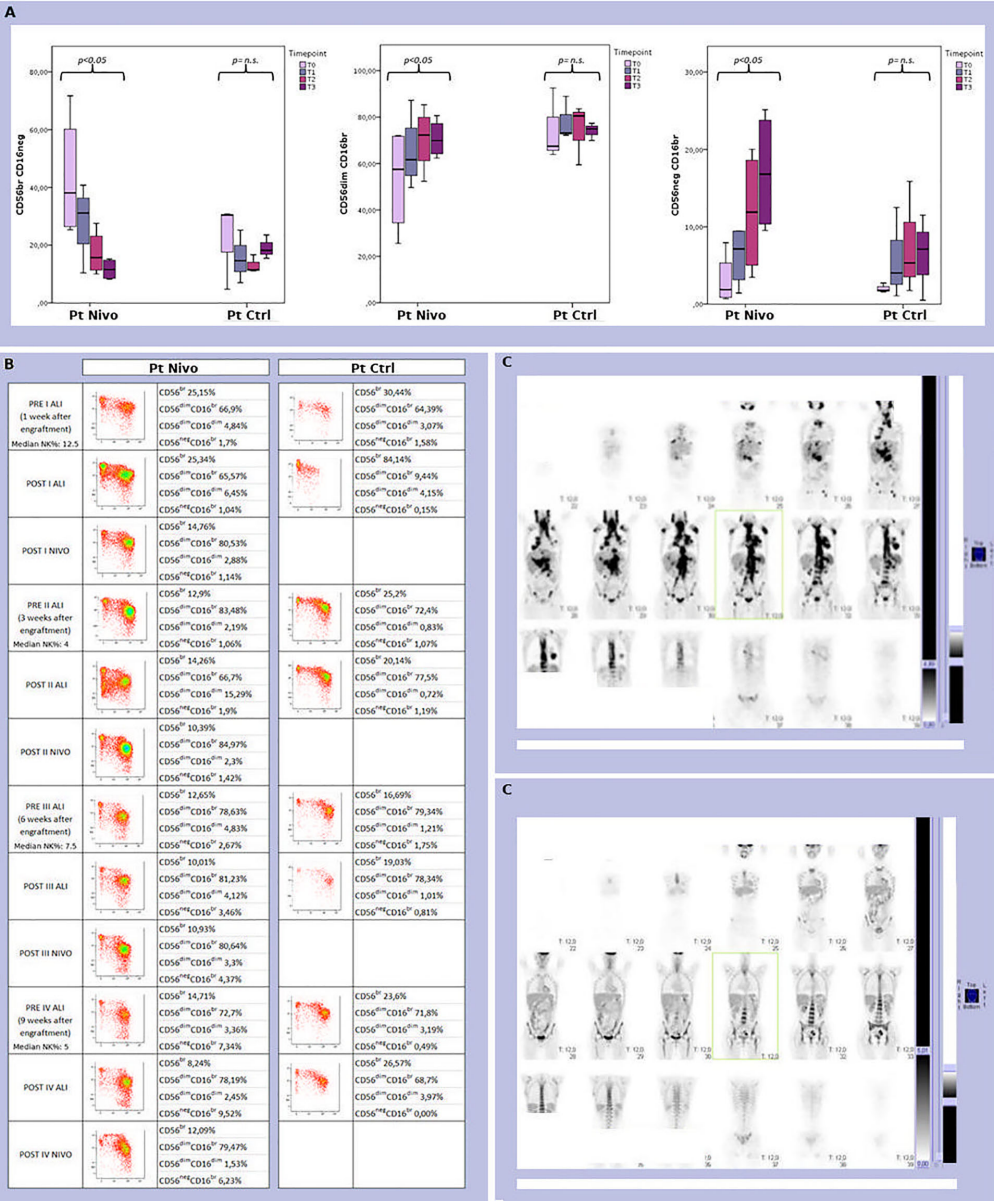
**(A)** Analysis of the size and distribution of CD56brightCD16neg/dim, CD56dimCD16bright, and CD56negCD16bright NK cell subsets derived from peripheral blood of 4 treated (Pt Nivo) and 3 control (Pt Ctrl) patients at different time points after ASCT (t0: pre-treatment; t1: first post ALI + nivolumab for treated patients and first ALI for control patient; t2: second post ALI + nivolumab for treated patients and second ALI for control patient; t3: third post ALI + nivolumab for treated patients and third ALI for control patients). Data represent the mean ± 95% CI. p < 0.05 **(B)** Comparison of peripheral blood NK cell subsets distribution in two patients representative of the two cohort -patient receiving ALI + nivolumab (Pt Nivo); patient receiving ALI alone (control) (Pt Ctrl)- at the different steps indicated in the Figure. ns, not significant. **(C)** PET scan from the patient receiving ALI + nivolumab at enrollment (upper panel **C**) and at end of treatment (EOT) (lower panel **C**). All the patients shown in **Figure 4**were seropositive for HCMV, but none of them experienced HCMV reactivation during all study period.

As expected, NK cells are the first lymphoid population emerging in the PB after ASCT. We analyzed the development of NK cells in all treated patients. The reciprocal expression of CD56 and CD16 by NK cells freshly isolated from 4 patients representative of the treated cohort, and 3 control patients, at different time points after transplantation is shown in **Figure 4A**. A representative example for each cohort is shown in **Figure 4B**. Interestingly, a faster expansion/reconstitution of NK cells displaying a highly differentiated surface phenotype was observed in the treatment cohort.

In detail, different patterns of NK cell development could be identified. Indeed, starting from one month after transplantation, in the group of patients undergoing ALI plus nivolumab, a relevant fraction of NK cells displayed a mature phenotype characterized by a CD56dimCD16bright expression (**Figure 4**). On the contrary, in control patients, NK cells were characterized by a more immature phenotype (high frequencies of CD56brightCD16neg/dim NK cells) even at late time points (three months) after transplantation (**Figure 4**). This event occurred in all the treated (ALI + nivolumab) patients analyzed, (**Figures 3** and **4A, B**). In addition, primarily in patients undergoing ALI + nivolumab, an aberrant and hyporesponsive subset of mature NK cells (namely CD56negCD16+), reminiscent of that described in patients with viremic HCMV/HIV, was detected (**Figures 4A, B**) (30, 31). Finally, PB from patients undergoing ALI + nivolumab was enriched in unconventional CD56dimCD16dim NK cells (**Figure 4A**). A phenotypically similar subset of NK cells, endowed with multifunctional activity (including potent killer and IFN-gamma producing capacity), was found in the bone marrow both of healthy children and of pediatric leukemic patients (32).

**Figure 5:**
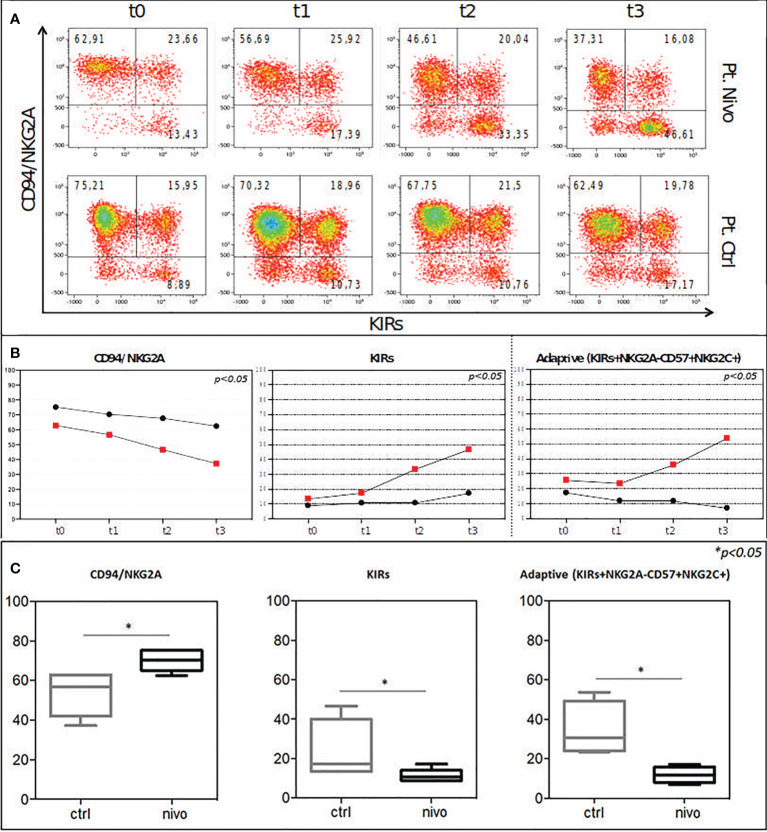
Peripheral blood NK cells were analysed for the expression of CD94/NKG2A in combination with KIRs post ASCT at different time points in treated patients (Pt Nivo) and control patients (Pt Ctrl) (t0: pre-treatment; t1: first post ALI + nivolumab for treated patients and first ALI for control patient; t2: second post ALI + nivolumab for treated patients and second ALI for control patient; t3: third post ALI + nivolumab for treated patients and third ALI for control patients). All the patients shown in **Figure 5** were seropositive for HCMV, but none of them experienced HCMV reactivation during all study period. **(A)** Two patients representative of the two cohort are shown. The percentage of NKG2A+KIR-, NKG2A+KIR+, and NKG2A-KIR+ NK-cell subsets/total NK cells are indicated in the corresponding quadrants at each time point. **(B)** NKG2A+ (Left), KIR+ (Center), and Adaptive (KIRs+NKG2A-CD57+NKG2C+) (Right) NK cells in two patients representative of the two cohort (red squares: Pt Nivo; black circles: Pt Ctrl) during each time point are shown. **(C)** The percentages of CD94/NKG2A+ (Left), KIR+ (Center), and Adaptive. (KIRs+NKG2A-CD57+NKG2C+) (Right) NK cells in treated (Pt Nivo) (n=3, black) and control (Pt Ctrl) (n=3, grey) HCMV seropositive patients are shown. Data represent the mean ± SD. *P < 0.05.

The reciprocal expression of CD94/NKG2A and KIRs by NK cells freshly isolated from two representative patients is shown in **Figure 5A**. One month after transplantation, CD94/NKG2A+ KIR- NK cells were largely predominant in all patients, whereas CD94/NKG2A- KIR+ NK cells were present in low proportions. An increase of CD94/NKG2A- KIR+ subset occurred primarily in treated patients starting from t2. At t3, the significant increase of CD94/NKG2A- KIR+ NK cells occurred in the treated group but not in the control group (**Figure 5**). This event occurred in all the treated (ALI + nivolumab) patients analyzed, regardless of their serological status for HCMV, but was more evident in treated patients who were seropositive for HCMV (**Figure 5** and data not shown).

Remarkably, PB from HCMV seropositive patients undergoing ALI + nivolumab became enriched over time in differentiated NK cells characterized by high expression of NKG2C receptor (the activating counterpart of NKG2A), as compared to HCMV+ control patients. This subset represents the so-called “adaptive” NK cell population that usually increases during HCMV infection/reactivation. In this case, however, this event occurred in the absence of HCMV reactivation, and only in HCMV seropositive patients treated with ALI + nivolumab (**Figures 5B, C**).

### Clinical Results

Early response assessment performed after cycle 2 of ALI + nivolumab showed a negative PET in 8/9 patients and a complete CT response in 5/9. EOT evaluation showed complete PET and CT response in all patients. Four patients were bridged to HSCT and are alive, in CR and free of graft-*versus*-host disease (GVHD) at the time of analysis. Notably, no excess acute or chronic GVHD was recorded among the patients who proceeded to HSCT after ALI + nivolumab.

Two of the remaining patients refused HSCT, one patient did not find a donor while two patients had recently completed the procedure. Overall, 8/9 R/R HL patients are alive and disease-free after a median follow-up of 28 months (95% IC 24.67-31.29 months, **Supplementary Figures S1, S2**). The promising CR rate compared to what expected with single agent nivolumab (10), alongside with the good tolerability of the procedure, prompted us to stop the feasibility study after patient #9 completed the treatment and to plan a multi-center phase II trial, with the aim of confirming the preliminary efficacy data in a larger number of patients.

Detailed therapeutic outcome of patients treated with ALI + nivolumab is provided in **Table 2**. Among the control group, 1/3 patients were in CCR while 2 patients relapsed 8 and 10 months after ASCT (median follow up 20 months, range 19-21).

**Table 2:** Therapy outcome after ALI/nivolumab.

	CT scan after cycle 2	PET scan after cycle 2	CT scan at EOT	PET scan at EOT	Allogeneic Stem-cell Transplant	Disease Status at Allogeneic SCT	Donor and Source of Allogeneic SCT	Disease and survival status at last FUP
**ALL PATIENTS (n=9)**	**PR: 3/9 PR CR: 6/9 CR**	**CR: 8/9**	**CR: 8/9**	**CR: 8/9**	**NO: 5/9**	**CR: 4/4**	**Haploidentical BM: 2/4**	**Alive and CR: 8/9**
					**YES: 4/9**		**HLA identical sibling BM: 2/4**	
**Patient #1**	CR	CR	CR	CR	YES	CR	Haploidentical, BM	Alive and CR
**Patient #2**	CR	CR	CR	CR	NO	–	–	Alive but relapsed
**Patient #3**	CR	CR	CR	CR	NO	–	–	Alive and CR
**Patient #4**	CR	CR	CR	CR	NO	–	–	Alive and CR
**Patient #5**	PR	CR	CR	CR	YES	CR	HLA-identical sibling, BM	Alive and CR
**Patient #6**	PR	CR	CR	CR	YES	CR	HLA-identical sibling, PB	Alive and CR
**Patient #7**	PR	CR	CR	CR	YES	CR	Haploidentical, BM	Alive and CR
**Patient #8**	CR	CR	CR	CR	NO	–	–	Alive and CR
**Patient #9**	PR	PR	CR	CR	NO	–	–	Alive in CR

CT: computed tomography; PET: positron emission tomography; EOT: end of treatment; SCT: stem cell transplantation; FUP: follow up; PR: partial remission, CR: complete remission, BM: bone marrow.

## Discussion

The results of this feasibility study suggest that early post-ASCT administration of nivolumab, supported by ALI, is safe and may be clinically effective in R/R HL patients. The combination of ALI and nivolumab may improve the results of either single anti-PD-1 therapy or ASCT performed in patients with active disease (5, 10, 11). Armand et al. reported the administration of pembrolizumab as post-ASCT consolidation in HL patients achieving at least PR before transplant, but considered at high risk of progression. In these patients the CI administration resulted in a significant improvement of PFS (33). In our study, we explored the clinical benefit of nivolumab in the worst setting of truly refractory HL patients that are usually not considered candidate for ASCT. The high risk of progression and the expected severe post-transplant immune suppression in these heavily pretreated patients, prompted us to plan early nivolumab supported by ALI (34). With the limitations due to the low number of patients in our trial, we may speculate that the striking activity in terms of CR rate may be explained by the rapid cytoreduction following ASCT conditioning that is able to induce the immunogenic death of HL cells, thus potentially enhancing the immune response triggered by CI therapy and ALI during the homeostatic reconstitution of lymphocyte repertoire. Indeed, post-ASCT ALI was very well tolerated and allowed a quick recovery of selected lymphocyte subsets. It has been widely reported that HSCT may provide a substantial contribute to cure advanced stage refractory HL patients if performed in CR (35). The high activity and the good tolerability of our strategy may therefore allow more refractory patients to enter CR and benefit from HSCT. In this view, 4 patients in our cohort did receive HCST in CR. However, regardless of subsequent HSCT, all enrolled patients except one maintained CR after discontinuation of immunotherapy, that was limited to 4 doses of nivolumab instead of the standard “until progression” schedule. Thus, the lower cumulative dose of nivolumab may reduce the risk of GVHD, which is very high in patients allotransplanted after CI therapy (35, 36).

The observation of a significant expansion of the mature NK compartment during ALI + nivolumab treatment suggests that NK cells may play a significant role in the anti-lymphoma response in this setting and is consistent with the observed trend for NK cell expansion reported by Armand and coworkers (33). Differently from what is observed in solid tumors, Reed Sternberg (RS) cells, albeit expressing PD-1 ligand, show HLA class I-II down-regulation, hampering the possibility of a cytotoxic T CD8+-mediated killing (10, 11, 15, 16). Indeed, it has been recently reported that not only T-lymphocytes, but also NK cells express the PD-1 receptor (9, 37). Taken together, these observations suggest a key role for NK cells in response to PD-1 blockade in HL, as these innate cells efficiently kill tumor cell targets not expressing HLA class I molecules. In addition, once activated, NK cells could release high amount of pro-inflammatory cytokines that can orchestrate other immune cell responses (38–40).

Here, we documented a quicker NK cell maturation in patients receiving both ALI and nivolumab, compared to the control cohort, despite the more severe degree of immune depression, due to the presence of active disease and the higher number of previous lines of chemo-immunotherapy received in comparison with the control group. On the basis of our data, it could be hypothesized that this setting may induce a strong imprinting to NK cell development by generating the uncommon CD56negCD16bright subpopulation (usually poorly represented in healthy condition), by accelerating the full maturation of NK cells (characterized by the CD94/NKG2A-KIR+CD57+ phenotype), and, in HCMV+ treated patients, by inducing the expansion of a subset of a terminally differentiated NK cell subset expressing NKG2C (the so-called adaptive NK cells), regardless of HCMV reactivation.

In physiological conditions, human NK cells include different cell subsets corresponding to different stages of NK cell differentiation (26). CD56bright CD16neg/dim NK cells (the major subset of NK cells in secondary lymphoid tissues) are considered as precursors of CD56dim CD16bright NK cells and have been usually considered “regulatory NK cells”. On the other end, CD56dim CD16bright NK cell population is the most represented in peripheral blood and is considered as the “cytotoxic population”. The terminally differentiated phenotype of CD56dim CD16bright NK cells is marked by the expression of CD57 (26, 40). Interestingly, unknown cofactors associated with HCMV infection may induce the generation of an additional type of fully mature NK cells characterized by the expression of the inhibitory receptor PD-1 (not necessarily co-expressed with NKG2C). These cells, called PD-1+ NK cells, show compromised effector functions against tumor cells expressing PD-1 ligands. Notably, this impaired antitumor NK cell activity can be partially restored by antibody-mediated disruption of PD-1/PD-L interaction (8, 39, 40).

Thus, our data support the notion that lymphocyte repletion and CI treatment early post-ASCT may accelerate the NK cell development/maturation and lead to a rapid accumulation of mature NK cells, which may exert anti-neoplastic activity as shown in the allo-transplant setting and following conventional immunosuppressive chemotherapy (41–44). In addition, we show that this setting may favor the expansion of the “adaptive” NK cell compartment in treated patients with a seropositivity status for HCMV, in the absence of HCMV reactivation. The “adaptive” NK cells that have been originally identified in HCMV+ individuals display some hallmarks of adaptive immunity, i.e., clonal expansion, longevity, as well as given epigenetic modifications and are characterized by more effective antitumor and antiviral immune responses, in terms of ADCC and IFN-γ production, features linked to changes in the expression of multiple intracellular proteins and transcription factors (39).

In conclusion, with the limitations of a small cohort of patients, our study shows that ALI + nivolumab is safe and feasible in R/R HL patients. Interestingly, the observed expansion and quick maturation of NK cells and, in some cases, the expansion of the “adaptive” NK cell compartment may suggest a role for NK cells in the response to CI in HL. Therefore, future studies should address the cytotoxic activity of mature NK cells and the correlation between biomarkers of NK cell maturation and function with clinical results.

## Data Availability Statement

The raw data supporting the conclusions of this article will be made available by the authors, without undue reservation.

## Ethics Statement

Ethical review and approval was not required for the study on human participants in accordance with the local legislation and institutional requirements. The patients/participants provided their written informed consent to participate in this study.

## Author Contributions

Project ideation, FG, PM, FB, and MaC. Methodology, MM, PM and MaC. Software, MF, AD, and RV. Validation, GZ. Formal analysis, FG, SP, MGr, MB, EM, and GZ. Investigation, MP, RiM, AB, MR, PC, and VA. Resources, MiC, ET, SC, AB, and VA. Data curation ET, NC, RoM, AS, and SL. Writing—original draft preparation, FG, PM, SP, MiC, and MF. Writing—review and editing, MM, JR, EM, GZ, and RL. Supervision, JR, EM, and RL. Project administration, EM and RL. Funding acquisition, MiC, SC, EM, and RL. All authors contributed to the article and approved the submitted version.

## Funding

This work was supported by grants from Compagnia di San Paolo (2019.866) to RL (Principal Investigator), EM (Group Leader Unige), FG, PM, SC, SP, MG, MB; Fondazione Associazione Italiana per la Ricerca sul Cancro (AIRC 5 × 1000-21147) to EM, SC, SP, MG; ROCHE 2017 to SP, EM. MGr was supported by a FIRC-AIRC fellowship for Italy.

## Conflict of Interest

The authors declare that the research was conducted in the absence of any commercial or financial relationships that could be construed as a potential conflict of interest.

## Publisher’s Note

All claims expressed in this article are solely those of the authors and do not necessarily represent those of their affiliated organizations, or those of the publisher, the editors and the reviewers. Any product that may be evaluated in this article, or claim that may be made by its manufacturer, is not guaranteed or endorsed by the publisher.
