# Supplementary material for: Post-Transplant Nivolumab Plus Unselected Autologous Lymphocytes in Refractory Hodgkin Lymphoma: A Feasible and Promising Salvage Therapy Associated With Expansion and Maturation of NK Cells
Source: Front Immunol. 2021 Nov 5;12:753890. doi: 10.3389/fimmu.2021.753890 (PMC8603402; doi:10.3389/fimmu.2021.753890)

## Supplementary Material

# Supplementary Data

**1.1 Lymphocyte Apheresis, Cryopreservation and Thawing**

The unstimulated lymphocyte apheresis procedure has been performed in steady state (e.g. patient must not have received any chemotherapy or steroids in the last 14 days) by processing a blood volume of about 5 litres, with a single-needle discontinuous flux machine.

The lymphocyte aphaeresis arrived to the Cell Factory from the Collection Center with attached complete blood count (CBC) of the product and one accompanying test tube. The test tube has been assessed for the CD3+ count at flow cytometry.

1 ml of heparin sodium was added to the product and, if necessary, the cell suspension has been dilute with physiological solution and 5% final of Human Albumin up to the final freezing volume. Volume has been calculated by maintaining a cell concentration equal to 50x10^6^/ml.

The cryoprotectant used is dimethyl sulfoxide(DMSO) 10% ; the product has been immediately transferred into 4-ml cryopreservation tubes and frozen at controlled-rate freezer (Ice cube SyLab).

After freezing procedures, samples have been stored in a liquid nitrogen cryo-container until the reinfusion, under temperature control with H24 remote alarms.

Blood cultures (BCs)for aerobic, anaerobic and fungi germs have been carried out on the final product.

The target aphaeresis quantity of CD3+ cell has been minimum of 5x10^8^/kg, if target dose has not been reached, a second unstimulated apheresis has been performed.

The thawing of the required dose of lymphocytes took place at the cryopreservation laboratory using a thermo stated bath at 37° C. In order to limit the DMSO toxicity, the thawed volume has been diluted 1: 1 with 0.9% Sodium chloride in a sterile environment. The reinfusion has always been performed within and no more than 15 minutes after thawing.

**1.2 Conditioning Chemotherapy**

ASCT conditioning has been performed according to conventional FEAM high dose chemotherapy (Fotemustine 150 mg/sqm days -7 and -6, Etoposide 200 mg/sqm + Cytarabine 400 mg/sqm days -5 to -2, Melphalan 140 mg/sqm day -1). A minimum of 3 x 10^6^/kg CD34+ autologous stem cells have been re-infused on day 0 in all patients. Granulocyte stimulating factor (G-CSF) has been administered from day 3 until the complete granulocyte recovery (i.e. ANC >2000/mmc). All patients achieved complete hematological engraftment after a median of 10 days (8-12).

2.1 Table I-S: T-Lymphocytes subpopulation definitions

|  |  | % CD8 cells in PB of healthy individuals | % leukocytes in healthy individuals  CD8% 10-20% lymph |
| --- | --- | --- | --- |
| Naïve (NA) | CD45RA+, CD62L+, CD27+, CD28+ | 16%-66% | 1,6-7 |
| Central Memory (CM) | CD45RAneg, CD62L+, CD27+, CD28+ | 1%-8% | 0,1-0,8 |
| EffectorMemory-CD27+ (EM-CD27+) | CD45Raneg, CD62L neg, CD27+, CD28+ (EM1) or CD28neg (EM2) | EM1 7-37%  EM2 0-4% | 0,7-4  0,1-0,4 |
| Effector Memory-CD27neg | CD45RAneg, CD62Lneg, CD27neg, CD28+ (EM4) or CD28neg (EM3) | EM3 0-7%  EM4 1-7% | 0,1-0,7  0,1-0,7 |
| EM RAINT | CD45RA+/-, CD62Lneg, CD27neg o basso, CD28neg | 2-18% | 0,2-1,8 |
| EM RA+ | CD45RA+, CD62Lneg, CD27+, CD28+ or CD28neg | 2-18% | 0,2-1,8 |
| Effectors (E) | CD45RA++, CD62Lneg, CD27neg, CD28neg | 2-53% | 0,2-5 |

2.2 Figure S1: Overall Survival in ALI +Nivolumab patients


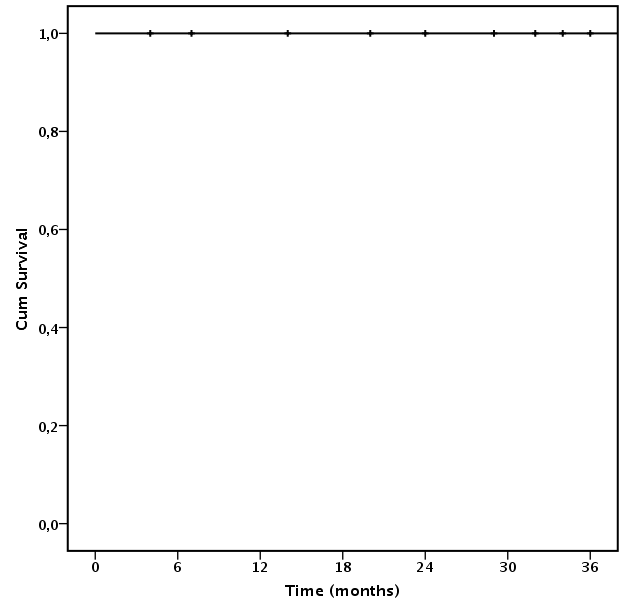


2.3 Figure S2: Progression-free survival in ALI +Nivolumab patients


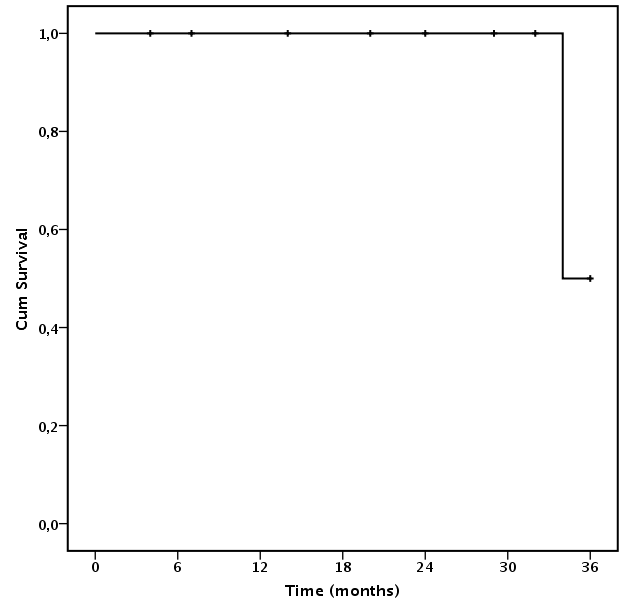

Supplement: Supplementary file 1 [file DataSheet_1.docx]
